# Adding concurrent chemotherapy to postoperative radiotherapy improves locoregional control but Not overall survival in patients with salivary gland adenoid cystic carcinoma—a propensity score matched study

**DOI:** 10.1186/s13014-016-0617-7

**Published:** 2016-03-22

**Authors:** Cheng-En Hsieh, Chien-Yu Lin, Li-Yu Lee, Lan-Yan Yang, Chun-Chieh Wang, Hung-Ming Wang, Joseph Tung-Chieh Chang, Kang-Hsing Fan, Chun-Ta Liao, Tzu-Chen Yen, Ku-Hao Fang, Yan-Ming Tsang

**Affiliations:** Department of Radiation Oncology, Chang Gung Memorial Hospital, Lin-Kou, No.5, Fu-Hsing ST., Kwei-Shan, Taoyuan, Taiwan, R.O.C; Department of Pathology, Linkou Chang Gung Memorial Hospital, Chang Gung University, Taoyuan, Taiwan, R.O.C; Department of Biostatistics and Informatics Unit, Clinical Trial Center, Linkou Chang Gung Memorial Hospital, Chang Gung University, Taoyuan, Taiwan, R.O.C; Department of Medical Imaging and Radiological Science, Chang Gung Memorial Hospital, Chang Gung University, Taoyuan, Taiwan, R.O.C; Department of Medical Oncology, Linkou Chang Gung Memorial Hospital, Chang Gung University, Taoyuan, Taiwan, R.O.C; Department of Otorhinolaryngology, Head and Neck Surgery, Linkou Chang Gung Memorial Hospital, Chang Gung University, Taoyuan, Taiwan, R.O.C; Department of Nuclear Medicine and Molecular Imaging Center, Linkou Chang Gung Memorial Hospital, Chang Gung University, Taoyuan, Taiwan, R.O.C; Department of Head and Neck Oncology Group, Linkou Chang Gung Memorial Hospital, Chang Gung University, Taoyuan, Taiwan, R.O.C; Graduate Institute of Clinical Medical Science, Chang Gung Memorial Hospital, Chang Gung University, Taoyuan, Taiwan, R.O.C; School of Traditional Chinese Medicine, Linkou Chang Gung Memorial Hospital, Chang Gung University, Taoyuan, Taiwan, R.O.C

**Keywords:** Salivary gland cancer, Chemoradiotherapy, Adenoid cystic carcinoma, Postoperative radiotherapy, Propensity score, Head and neck

## Abstract

**Purpose:**

To compare the long-term outcomes in patients with salivary gland adenoid cystic carcinoma (SGACC) treated with post-operative chemoradiotherapy (POCRT) *versus* post-operative radiotherapy (PORT).

**Methods:**

We retrospectively reviewed the records of 91 SGACC patients treated with surgery followed by PORT (*n* = 58) or POCRT (*n* = 33) between 2000 and 2013. Treatment outcomes between groups were compared using propensity score matching (1:1 nearest neighbor).

**Results:**

The median radiation dose was 66 Gy, and patients were followed up for a median of 71 months. Cisplatin-based concurrent regimens were the most commonly used chemotherapy schedules. In the entire study cohort, patients undergoing POCRT showed a trend toward higher locoregional control (LRC) rates than those treated with PORT alone at both 5 and 8 years (97 and 97 % *versus* 84 and 79 %, respectively; *P* = .066). Distant metastases were the most common form of treatment failure and occurred in 31 (34 %) patients (PORT, *n* = 17; POCRT, *n* = 14). After propensity score matching (33 pairs), patients receiving POCRT had 5- and 8 year LRC rates of 97 and 97 %, respectively, compared with 79 and 67 % for patients treated with PORT alone (*P* = .017). The two groups did not differ significantly in terms of distant metastasis-free survival (DMFS), disease-free survival (DFS), and overall survival (OS). However, a significantly better opioid-requiring pain-free survival (ORPFS) was achieved in POCRT group (*P* = .038). Subgroup analyses revealed that patients with stage III − IV disease (*P* = .040 and .017), positive surgical margins (*P* = .011 and .050), or perineural invasion (*P* = .013 and .035) had significantly higher 5- and 8 year LRC and ORPFS when treated with POCRT, respectively.

**Conclusions:**

In SGACC patients, adding concurrent chemotherapy to PORT may increase LRC and ORPFS rates, particularly in presence of stage III − IV disease, positive surgical margins, or perineural invasion. However, no significant differences in DMFS, DFS, and OS were observed.

## Introduction

Despite being the most common form of salivary gland cancer, salivary gland adenoid cystic carcinoma (SGACC) is a rare malignancy which accounts for only 1 % of all head and neck tumors [1, 2]. Because of its local invasiveness, post-operative radiotherapy (PORT) is frequently used to increase locoregional control (LRC), particularly in patients bearing adverse prognostic factors, including positive surgical margins, advanced T stage, and perineural invasion (PNI) [3–5]. Despite important advances in combination therapies, the 10 year rate of locoregional recurrence continues to remain high (~30 %) [3–5]. Several multicenter randomized trials have demonstrated the effectiveness of post-operative chemoradiotherapy (POCRT) in patients with head and neck squamous cell carcinoma (HNSCC) [6–8]. However, as a result of the rarity of SGACC, data concerning the role of adjuvant therapies in this clinical entity remain scarce. Although chemotherapy is generally reserved for the treatment of recurrent or metastatic SGACC [9], the clinical usefulness of adding concurrent chemotherapy to PORT remains unclear.

Based on the evidence supporting the clinical utility of POCRT in HNSCC patients [6–8], we have been utilizing POCRT in our SGACC patients bearing adverse prognostic factors. In the present study, we retrospectively investigated the long-term outcomes of SGACC patients who were treated with surgery followed by PORT or POCRT in our institutions. Treatment outcomes between the PORT and POCRT groups were compared using propensity score matching in order to minimize biases. We also performed subgroup analyses with the goal of identifying specific SGACC patients who can benefit most from POCRT.

## Materials and method methods

### Patients and clinical work-up

The study complied with the tenets of the Declaration of Helsinki and the research protocol was approved by the local Institutional Review Board (102-0938B). We retrospectively reviewed the records of 174 SGACC patients who were treated in the Linkou, Keelung, and Chiayi Chang Gung Memorial Hospitals between January 2000 and December 2013. After the exclusion of patients with unresected tumors (*N* = 24), distant metastases (*N* = 23), synchronous cancers (*N* = 4), a history of previous radiotherapy (RT) in the head and neck area (*N* = 6), and no adjuvant RT after surgery (*N* = 26), a total of 91 patients who received either PORT or POCRT were included in the final analysis.

The pretreatment work-up and the follow-up schedules were in accordance with the general guidelines for HNSCC patients as previously described [10]. Patient staging was performed according to the seventh edition (2010) of the American Joint Committee on Cancer (AJCC) Staging System. All of the pathological specimens were reviewed by experienced head and neck pathologists according to the 2005 World Health Organization (WHO) histological classification. Treatment-related adverse events were graded using the Common Terminology Criteria for Adverse Events (CTCAE; version 3.0) [11].

### Treatment

All of the treatment decisions were taken by consensus of multidisciplinary Head and Neck Tumor Boards. Neck dissection was performed in patients with clinically positive nodes or locally advanced tumors. The study patients received surgical treatment with curative intent followed by either PORT (*N* = 58) or POCRT (*N* = 33). All of the patients received megavoltage X-ray irradiations via three-dimensional conformal radiotherapy (3D-CRT), intensity-modulated radiation therapy (IMRT), or volumetric-modulated arc therapy (VMAT, RapidArc) delivery systems. The prophylactic irradiation dose was 46–50 Gray (Gy), with a 60–66 Gy boost to high-risk areas (dose per fraction: 1.8–2 Gy, given five times per week). Over the last years, there was an increasing use of chemoradiotherapy in our patient population. In general, chemotherapy was administered in patients with advanced disease or in presence of adverse pathological risk factors [6, 7, 10]. Cisplatin-based concurrent regimens were the most commonly used chemotherapy schedules. Patients typically received cisplatin at 100 mg/m^2^ once every 3 weeks or 40 mg/m^2^ once per week [12].

### Statistical analysis

Intergroup differences in continuous variables were tested using independent Student’s *t*-tests. Categorical data were compared using the Pearson’s chi-squared test or the Fisher’s exact test, as appropriate. LRC, distant metastasis-free survival (DMFS), disease-free survival (DFS), and overall survival (OS) were calculated from the date of surgery to the date of the events of interest. Opioid-requiring pain-free survival (ORPFS) was calculated based on the date of the first prescription of opioids for the relief of any pain occurring 6 months after adjuvant therapy. Survival curves were plotted using the Kaplan-Meier method and compared using the log-rank test. The propensity score was estimated using logistic regression, with the dependent variable being treatment with POCRT. All data were analyzed using the SPSS 20.0 software package (IBM Corporation, Armonk, NY, USA). Propensity score matching was performed using the MatchIt package of the R program, version 2.12.0 (R Foundation for Statistical Computing, Vienna, Austria).

## Results

### Patient characteristics

The general characteristics of the study patients are summarized in Table 1. Patients who were treated with POCRT were characterized by a significantly higher prevalence of positive surgical margins (defined as any resection margin width of less than 1 mm; *P* = .032) and nodal extracapsular spread (ECS) (*P* = .022), a significantly longer time interval between surgery and RT (*P* = .018) and trends toward higher T stage (*P* = .099), larger tumor size (*P* = .051) and higher bone invasion rate (*P* = .066). Compared with patients who received PORT, those treated with POCRT were also more likely to receive magnetic resonance imaging (MRI) (*P* = .044), ^18^F-fluorodeoxyglucose positron emission tomography (^18^F-FDG-PET; *P* = .039), IMRT/VMAT (*P* < .001), and having been treated with RT more recently (2010–2013 *versus* 2005–2009 *versus* 2000–2004; *P* = .001).

**Table 1:** General characteristics of the study patients

		Entire Cohort			Propensity Score Matching		
Characteristic		PORT (*N* = 58)	POCRT (*N* = 33)	*P*	PORT (*N* = 33)	POCRT (*N* = 33)	*P*
Age (years)	Mean	50 ± 15	51 ± 12	.793	48 ± 15	51 ± 12	.372
Sex	Female/Male	37 (64)/21 (36)	17 (52)/16 (49)	.275	18 (55)/15 (46)	17 (52)/16 (49)	1.000
Tumor subsite	Parotid	16 (28)	6 (18)	.394	7 (21)	6 (18)	.692^a^
	Submandibular	16 (28)	8 (24)		11 (33)	8 (24)	
	Sublingual	8 (14)	3 (9)		4 (12)	3 (9)	
	Minor salivary	18 (31)	16 (49)		11 (33)	16 (49)	
Performance score	0-1/2	57 (98)/1 (2)	30 (91)/3 (9)	.134^a^	33 (100)/0 (0)	30 (91)/3 (9)	.238^a^
T stage	T1	23 (40)	5 (15)	.099	12 (36)	5 (15)	.137^a^
	T2	15 (26)	10 (30)		9 (27)	10 (30)	
	T3	5 (9)	5 (15)		1 (3)	5 (15)	
	T4	15 (26)	13 (39)		11 (33)	13 (39)	
Tumor size (cm)	Mean	2.4 ± 1.3	3.1 ± 1.7	.051	2.4 ± 1.3	3.1 ± 1.7	.060
N stage	N0	52 (90)	26 (79)	.332^a^	31 (94)	26 (79)	.228^a^
	N1	3 (5)	3 (9)		1 (3)	3 (9)	
	N2	3 (5)	4 (12)		1 (3)	4 (12)	
Disease stage	I	21 (36)	4 (12)	.103	11 (33)	4 (12)	.200^a^
	II	14 (24)	10 (30)		8 (24)	10 (30)	
	III	6 (10)	5 (15)		2 (6)	5 (15)	
	IV	17 (29)	14 (42)		12 (36)	14 (42)	
MRI		17 (29)	17 (52)	.044	10 (30)	17 (52)	.132
^18^F-FDG-PET		15 (26)	16 (49)	.039	15 (46)	16 (49)	1.000
Surgical margins	<1 mm/≥1 mm	38 (66)/20 (35)	28 (85)/5 (15)	.032	26 (79)/7 (21)	28 (85)/5 (15)	.751
Histological features	Perineural invasion	38 (66)	27 (82)	.147	24 (73)	27 (82)	.558
	Nodal ECS	1 (2)	5 (15)	.022^a^	0 (0)	5 (15)	.053^a^
	Bone invasion	9 (16)	11 (33)	.066	8 (24)	11 (33)	.587
	Skin invasion	2 (3)	0 (0)	.533^a^	1 (3)	0 (0)	1.000^a^
	Muscle invasion	19 (33)	7 (21)	.335	11 (33)	7 (21)	.408
	Lymphatic invasion	5 (9)	4 (12)	.718^a^	1 (3)	4 (12)	.355^a^
	Vascular invasion	2 (3)	3 (9)	.349^a^	2 (6)	3 (9)	1.000^a^
RT technique	3D-CRT	30 (52)	3 (9)	< .001	8 (24)	3 (9)	.340
	IMRT	19 (33)	20 (61)		17 (52)	20 (61)	
	VMAT	9 (16)	10 (30)		8 (24)	10 (30)	
RT dose	>66 Gy	13 (22)	6 (18)	.678^a^	5 (15)	6 (18)	.810^a^
	60–66 Gy	41 (71)	26 (79)		25 (76)	26 (79)	
	<60 Gy	4 (7)	1 (3)		3 (9)	1 (3)	
RT period	2010–2013	13 (22)	12 (36)	.001	11 (33)	12 (36)	.556
	2005–2009	15 (26)	17 (52)		11 (33)	17 (52)	
	2000–2004	30 (52)	4 (12)		11 (33)	4 (12)	
Neck treatment	Elective	27 (47)	15 (46)	.677	13 (39)	15 (46)	.411
	Therapeutic	8 (14)	7 (21)		4 (12)	7 (21)	
	None	23 (40)	11 (33)		16 (49)	11 (33)	
Treatment time	Surgery to RT (days)	31 ± 12	37 ± 11	.018	35 ± 11	37 ± 11	.544
	RT duration (days)	47 ± 9	48 ± 4	.770	46 ± 6	48 ± 4	.189

Numbers in parentheses indicate percentages. *Abbreviations*: *RT* radiotherapy, *PORT* postoperative radiotherapy, *POCRT* postoperative chemoradiotherapy, *MRI* magnetic resonance imaging, ^*18*^ *F*-*FDG PET* ^18^F-Fluorodeoxyglucose positron emission tomography, *ECS* extracapsular spread, *3D*-*CRT* 3-dimensional conformal radiotherapy, *IMRT* intensity-modulated radiotherapy, *VMAT* RapidArc volumetric-modulated arc therapy. ^a^Fisher’s exact test

The median cumulative radiation dose for the entire study cohort was 66.0 Gy (range: 7.2–76.0 Gy). Elective neck treatments including neck dissection (*N* = 13) and/or irradiation (*N* = 41) were applied to 42 patients. We found no statistically significant differences in terms of radiation dose, neck treatment modality, and duration of radiotherapy between the PORT and POCRT groups (Table 1).

Intravenous cisplatin-based concurrent chemotherapy was administered to 31 (94 %) of the 33 POCRT patients. Among them, a single agent was given to 22 patients, whereas concurrent oral uracil-tegafur and cisplatin were used in nine patients. The median cumulative cisplatin dose was 200 mg/m^2^. Twenty-four (77 %) of the 31 patients completed their planned chemotherapy course. Non-cisplatin-based regimens consisted of either single-agent mitomycin C (*N* = 1) or cetuximab (*N* = 1). Neither neoadjuvant nor adjuvant chemotherapy were performed, the only exception being seven patients who received one or two courses of chemotherapy during the radiotherapy waiting time.

### Treatment outcomes

At the end of the study, 65 (71 %) patients were alive (median follow-up time for survivors: 71 months; range: 8–186 months). Disease-specific deaths occurred in 20 patients (PORT, *N* = 14; POCRT, *N* = 6), and 19 of them expired because of distant metastases. Only one patient in the PORT arm died of local tumor progression. Death caused by intercurrent diseases occurred in 6 study participants (PORT, *N* = 4; POCRT, *N* = 2). The 5- and 8 year OS rates in the entire cohort were 83 and 71 %, respectively. In addition, ten (11 %) patients developed secondary malignancies (PORT, *n* = 7; POCRT, *n* = 3), including gastric (*n* = 3), pancreatic (*n* = 1), rectal (*n* = 1), ovarian (*n* = 1), and pulmonary (*n* = 1) adenocarcinomas, gastrointestinal stromal tumor (*n* = 1), cholangiocarcinoma (*n* = 1), and cervical squamous cell carcinoma (*n* = 1).

Recurrences were observed in 32 (35 %) patients, and the 5- and 8 year DFS rates were 64 and 56 %, respectively. Distant metastases were the most common form of treatment failure and occurred in 31 (34 %) patients after a median time of 34 months (range: 5–128). The 5- and 8 year DMFS rates were 65 and 59 %, respectively. Eleven (12 %) patients showed locoregional relapse (median time: 27 months, range: 5–72 months), and eight of them were found to occur within high radiation dose regions. The detailed characteristics of patients who developed locoregional recurrences are summarized in Table 2. The 5- and 8 year cumulative LRC rates were 89 and 85 %, respectively.

**Table 2:** Baseline characteristics of the 11 patients with salivary gland adenoid cystic carcinoma who developed locoregional recurrences after adjuvant therapy

Case	Tumor location	pT	pN	Stage	M	ECS	PNI	BI	SI	MI	LI	VI	LR	RR	DM	Arm
1	Parotid gland	4	0	IVa	+	-	+	-	-	+	-	-	+ (in-field)	-	+	PORT
2	Parotid gland	2	0	II	+	-	+	-	-	-	-	-	+ (in-field)	+ (in-field)	+	PORT
3	Hard palate	4	1	IVa	+	+	+	+	-	+	-	-	+ (in-field)	-	+	POCRT
4	Hard palate	4	0	IVa	+	-	+	+	-	+	-	-	+ (in-field)	-	+	PORT
5	Nasal cavity	4	0	IVa	+	-	+	+	-	+	-	-	+ (in-field)	-	+	PORT
6	Gingivae	4	0	IVa	+	-	+	+	-	-	-	+	+ (in-field)	-	+	PORT
7	Submandibular gland	1	2b	IVa	+	-	+	-	-	+	-	-	+ (in-field)	+ (in- & out-field)	-	PORT
8	Submandibular gland	4	0	IVa	+	-	+	-	-	+	-	-	-	+ (in-field)	+	PORT
9	Submandibular gland	2	0	II	+	-	+	-	-	-	-	-	-	+ (out-field)	+	PORT
10	Submandibular gland	3	1	III	+	-	+	-	-	+	+	-	-	+ (out-field)	+	PORT
11	Submandibular gland	4	0	IVa	+	-	+	-	-	+	-	-	-	+ (out-field)	+	PORT

*Abbreviations*: *pT* pathological T stage, *pN* pathology *N stage*, *M* surgical margins, *PNI* perineural invasion, *BI* bone invasion, *SI* skin invasion, *MI* muscle invasion, *LI* lymphatic invasion, *VI* vascular invasion, *LR* local recurrence, *RR* regional node recurrence, *DM* distant metastases, *PORT* postoperative radiotherapy, *POCRT* postoperative chemoradiotherapy

Fourteen of the 32 patients with cancer recurrence received salvage treatment (surgery, *N* = 6; radiotherapy, *N* = 6; chemotherapy, *N* = 6), but none of them was successfully rescued. The best supportive care was provided to the remaining patients. However, several years of life expectancy were observed in patients after a diagnosis of cancer relapse. The median survival time was 29 months (range: 7–108 months) for patients with locoregional recurrence and 25 months (range: 1–98 months) for cases with distant metastases, respectively. Twelve patients suffered from cancer-related pain requiring continuous long-term opioid analgesics. The 5- and 8 year ORPFS rates were 91 and 83 % for the entire cohort, respectively. In addition, the majority of patients with locoregional failures experienced moderate-to-severe symptoms, including cosmetic disfiguration (*N* = 9), chronic ulceration (*N* = 6), soft tissue infections requiring antibiotic treatment and hospitalization (*N* = 6), numbness, paresthesia, or cranial nerve palsy (*N* = 9), opioid-requiring pain (*N* = 8), and anxiety or insomnia requiring drug therapy (*N* = 7).

### Comparisons of POCRT and PORT

**Fig. 1:**
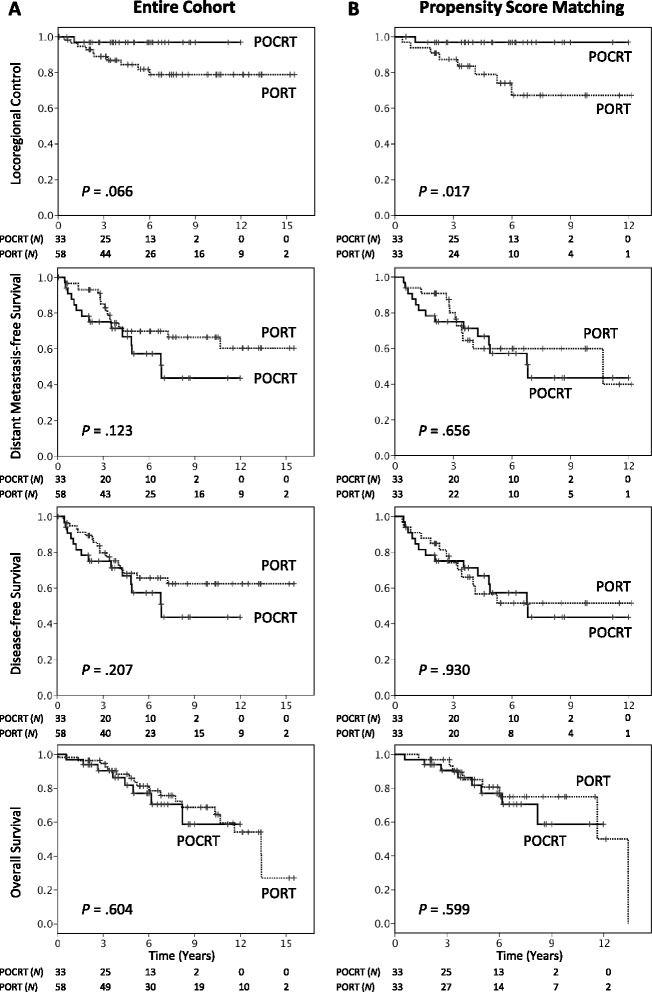
Locoregional control, distant metastases-free survival, disease-free survival, and overall survival rates in patients with salivary gland adenoid cystic carcinoma treated with postoperative chemoradiotherapy (POCRT) (solid lines) or postoperative radiotherapy (PORT) (dashed lines), before (**a**) and after (**b**) propensity score matching

**Fig. 2:**
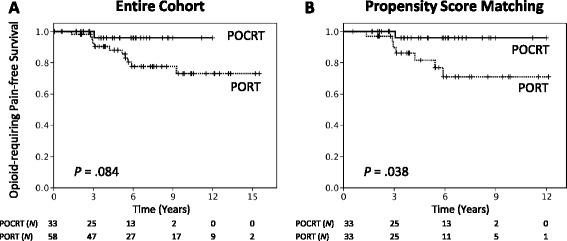
Opioid-requiring pain-free survival in patients with salivary gland adenoid cystic carcinoma treated with post-operative chemoradiotherapy (POCRT) (solid lines) or post-operative radiotherapy (PORT) (dashed lines), before (**a**) and after (**b**) propensity score matching

In the entire study cohort, a trend toward higher LRC was identified in patients who received POCRT than those who were treated with PORT alone at both 5 and 8 years (97 and 97 % *versus* 84 and 79 %, respectively; *P* = .066, Fig. 1a). We also noted a trend toward better 5- and 8 year ORPFS rates in POCRT patients than in the PORT group (96 and 96 % *versus* 88 and 78 %, *P* = .084, Fig. 2a). However, no statistically significant differences were observed between the two groups in terms of DMFS, DFS, and OS (Fig. 1a).

To minimize the inherent selection biases in our retrospective cohort, we used propensity score matching to balance the PORT and POCRT groups. To this aim, we focused on preexisting significant differences at the *P* ≤ 0.1 level (Table 1). We observed that higher disease stages were significantly correlated with higher T stages (*P* < .001), larger tumor size (*P* < .001), presence of nodal ECS (P = .008), bone invasion (*P* < .001), and use of MRI (*P* < .001). Moreover, patients who received modern RT techniques were more likely to be treated in recent RT periods (*P* < .001) and receive ^18^F-FDG-PET (*P* < .001). Therefore, we selected disease stage, RT technique, margin status, and interval from surgery to RT as independent variables for propensity score calculation. Patients were matched in a 1:1 ratio using the nearest-neighbor method, without replacement. Finally, a total of 33 patient pairs were examined to estimate the potential usefulness of adding concurrent chemotherapy to PORT.

After propensity score matching, all of the preexisting statistical differences between groups were well-balanced, the only exceptions being trends toward a larger tumor size (*P* = .060) and a higher prevalence of nodal ECS (*P* = .053) in the POCRT group (Table 1). The median follow-up period was 70 months for patients who received POCRT and 68 months for patients treated with PORT. Patients who were treated with POCRT had 5- and 8 year LRC rates of 97 and 97 %, respectively, compared with 79 and 67 % for patients who received PORT alone (*P* = .017, Fig. 1b). No significant differences between the POCRT and PORT groups were observed in terms of DMFS, DFS, and OS (Fig. 1b). However, significantly better 5- and 8 year ORPFS rates were achieved in POCRT patients than in the PORT group (96 and 96 % vs 82 and 71 %, *P* = .038, Fig. 2b).

**Fig. 3:**
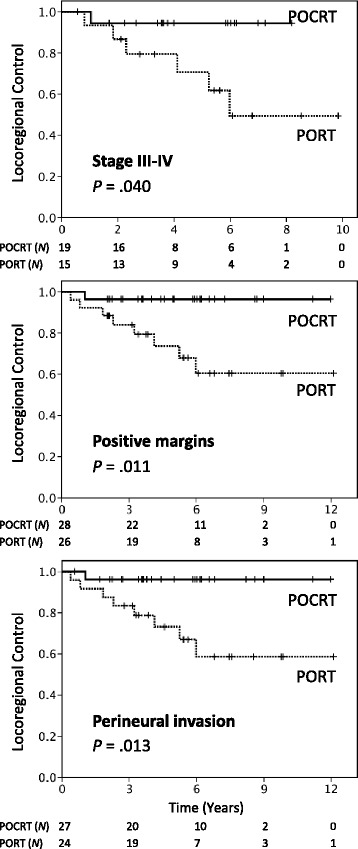
Locoregional control curves of patients with salivary gland adenoid cystic carcinoma with stage III-IV disease, positive resection margins, or perineural invasion treated with postoperative chemoradiotherapy (POCRT) (solid lines) or postoperative radiotherapy (PORT) (dashed lines) in the propensity score-matched cohort

We performed subgroup analyses with the goal of identifying specific subgroups of SGACC patients who can benefit most from POCRT. To this aim, the pathological parameters were separately examined in the propensity score-matched cohort (33 patient pairs). The results indicated that SGACC patients with positive surgical margins (*P* = .011 and .050), PNI (*P* = .013 and .035), or stage III − IV disease (*P* = .040 and .017) had significantly higher 5- and 8 year LRC and ORPFS rates when treated with POCRT, respectively (Table 3, Fig. 3). However, again we did not find significant differences between the PORT and POCRT groups in terms of DMFS, DFS, and OS following stratification for these parameters. Subgroup analyses of patients with ECS, skin invasion, and invasion to the lymphatics or vascular space were not meaningful because of small sample sizes.

**Table 3:** Treatment outcomes of patients with salivary gland adenoid cystic carcinoma bearing adverse pathological risk factors in the propensity score-matched cohort, treatment with PORT *versus* POCRT

		LRC			DMFS			DFS			OS			ORPFS		
Variable	*N*	5-yr	8-yr	*P*	5-yr	8-yr	*P*	5-yr	8-yr	*P*	5-yr	8-yr	*P*	5-yr	8-yr	*P*
Stage III-IV																
PORT	15	70.6	49.4	.040	47.7	47.7	.601	40.9	32.7	.963	77.8	60.0	.705	62.2	43.6	.017
POCRT	19	94.4	94.4		43.8	29.2		43.8	29.2		75.9	60.7		92.9	92.9	
Margins < 1 mm																
PORT	26	73.7	60.5	.011	59.0	59.0	.669	54.3	48.3	.972	86.2	74.1	.894	81.6	68.2	.050
POCRT	28	96.4	96.4		54.5	37.4		54.5	37.4		79.8	71.8		95.5	95.5	
Perineural invasion																
PORT	24	73.1	58.6	.013	54.5	54.5	.849	49.9	43.7	.857	81.6	69.2	.792	77.2	63.4	.035
POCRT	27	96.2	96.2		52.1	41.7		52.1	41.7		77.2	68.7		95.0	95.0	
Bone invasion																
PORT	8	87.5	52.5	.425	45.0	45.0	.652	45.0	30.0	.851	72.9	72.9	.220	72.9	43.8	.260
POCRT	11	90.0	90.0		50.5	50.5		50.5	50.5		52.6	52.6		83.3	83.3	
Muscle invasion																
PORT	11	57.3	43.0	.240	54.5	54.5	.260	43.0	28.6	.608	77.9	48.7	.772	54.5	40.9	.214
POCRT	7	85.7	85.7		19.0	19.0		19.0	19.0		80.0	80.0		83.3	83.3	

Data for survival estimates are expressed as percentages

*Abbreviations*: *PORT* postoperative radiotherapy, *POCRT* postoperative chemoradiotherapy, *LRC* locoregional control, *DMFS* distant metastasis-free survival, *DFS* disease-free survival, *OS* overall survival, *ORPFS* opioid-requiring pain-free survival

#### Treatment-related complications

Acute grade 0–2 and grade 3 mucositis occurred in 68 and 32 % of patients in the PORT group, respectively, as compared with 64 and 36 % in the POCRT arm (*P* = .778). Grade 0–2 and grade 3 dermatitis were observed in 94 and 6 % of patients in the PORT group, respectively, as compared with 92 and 8 % in the POCRT arm (*P* = .859). In the POCRT group, grade 0–2 and grade 3–4 hematological toxicities were identified in 77 and 23 % of patients, respectively. Grade 0–1 and grade 2 xerostomia at 1 year of follow-up developed in 63 and 37 % of patients in the PORT group, respectively, as compared with 81 and 19 % in the POCRT arm (*P* = .433).

## Discussion

Despite its generally indolent course, locoregional recurrence and distant metastases remain major clinical issues in SGACC patients who bear adverse prognostic factors even when aggressive treatment modalities are used [3–5, 13]. In patients with locally advanced HNSCC, POCRT has been shown to confer survival benefits and has been incorporated into the current standards of care [6, 7]. However, the question as to whether the addition of concurrent chemotherapy may improve outcomes in SGACC patients undergoing PORT remains open. Accordingly, the available data on the clinical utility of POCRT in patients with salivary gland cancers remain scarce and controversial. In a matched case–control study of salivary gland cancer patients who were treated either with platinum-based POCRT (*N* = 12) or PORT alone (*N* = 12), a significantly higher 3 year OS was observed in the POCRT arm (83 % vs 44 %) after a short follow-up period (median: 14.9 months for PORT and 31.6 months for POCRT) [14]. In another retrospective study of 35 patients undergoing adjuvant IMRT either with (*N* = 22) or without (*N* = 13) concurrent chemotherapy, no clear superiority for concurrent chemoradiation was observed after a median follow-up time of 2.3 years [15]. Because these studies included a markedly high number of different histological subtypes with small simple sizes and short follow-up periods, the question as to whether their findings can be generalizable to SGACC remains open. Because of the slowly progressive nature of SGACC, the median time to recurrence following adjuvant treatment is approximately 2–3 years [4, 5] making long-term follow-up particularly important to obtain reliable data.

In this study, we described the long-term outcomes of a multicenter series of SGACC patients treated with either POCRT or PORT. Besides performing a central review of all pathological specimens, we used propensity score matching to minimize the potential bias associated with a retrospective analysis. The results of our study demonstrate that SGACC patients treated with POCRT have significantly better LRC rates than those treated with PORT (5- and 8 year; 97 and 97 % *versus* 79 and 67 %, *P* = .017, Fig. 1b), particularly in presence of positive resection margins, PNI, or stage III − IV disease (Table 3). These findings indicate that concomitant chemotherapy has the potential to produce substantial radiosensitization of SGACC. Only a limited number of published studies have investigated the feasibility of chemoradiotherapy in SGACC patients, with the majority of them having been focused on the potential usefulness of definitive chemoradiotherapy. A case series of five non-resected SGACC patients managed with definitive carboplatin and paclitaxel-based chemoradiotherapy showed no locoregional recurrences after a median follow-up of 36 months, although one patient developed distant metastases at 7 months [16]. In a retrospective study that included 16 patients with locally advanced SGACC, the use of definitive chemoradiotherapy (with either intra-arterial or intravenous cisplatin or carboplatin) was associated with a 5 year local progression-free survival rate of 61 % after a median follow-up of 61 months [17]. In this report, distant metastasis was the most common pattern of treatment failure (five patients), and the 5 year OS and progression-free survival rates were 87 and 39 %, respectively. Taken together, these data suggest that platinum-based chemoradiation can exert a significant local cancer killing effect in SGACC.

In accordance with the published literature [3–5, 17], distant metastasis was the main pattern of treatment failure and the most common cause of disease-specific mortality in our study. Although our findings indicate that the addition of concurrent chemotherapy to PORT significantly reduced locoregional relapses, we did not observe a decrease of hematogenous spread among these patients. Patients bearing multiple adverse prognostic factors were still at high risk of distant failure even with controlled locoregional disease. In addition, because of the slowly progressive course of SGACC, the median survival in patients with recurrence was reported to be as long as 2–3 years [18]. In our study cohort, the median survival time after a diagnosis of locoregional recurrence was 29 months (range: 7–108 months). These observations potentially explain the fact that the LRC benefit resulting from POCRT did not translate into a survival advantage.

Because the goals of comprehensive cancer care are not limited to maximize patient survival but also improve quality of life, we believe that the significant reduction in the rate of locoregional failure in SGACC patients should be considered clinically significant (especially because this type of failure is generally unsalvageable and symptomatic). Patients with locoregional relapses have several years of life expectancy but are particularly prone to symptoms that can seriously affect the overall quality of life (e.g., intractable pain). The time to first use of opioid analgesics is a meaningful clinical endpoint in the field of oncology and has been validated for assessing treatment efficacy in a number of previous prospective randomized trials [19–22]. Intriguingly, propensity score analysis demonstrated that POCRT was significantly associated with a higher ORPFS (5 year: 96 % *versus* 82 %, *P* = .038, Fig. 2b). Subgroup analyses also demonstrated ORPFS improvement in patients with positive resection margins, PNI, or stage III − IV disease. These results suggest that an increase in LRC rates may be paralleled by a reduced risk of cancer-related pain (Table 3).

Despite every effort made to perform a very careful review of our data and multiple statistical examinations, there are still some unavoidable limitations inherent to our study. They include, but are not limited to, the non-randomized retrospective nature of the research and the long enrollment period. The relatively small number of events in this observational cohort and several unmeasured factors (including subjective treatment decisions and the varying surgical expertise over time) may have biased our results. In addition, we cannot exclude an underestimation of treatment-related side effects in patients who received concurrent chemotherapy in addition to postoperative radiotherapy. In this regard, several prospective randomized trials conducted in patients with head and neck malignancies have demonstrated that adjuvant chemoradiation can increase grade III − IV acute toxicities as compared with PORT alone (EORTC 22931: 41 % vs 21 %, *P* = 0.001; RTOG 9501: 77 % vs 34 %, *P* < 0.001) [6, 7] with an insignificant higher incidence of grade III − V late complications (RTOG 9501/intergroup long-term report: 24.9 % vs 20.5 %, *P* = 0.34) [23]. We are also aware that a median follow-up of 71 months is relatively short for the assessment of survival outcomes in patients with a slowly progressive malignancy like SGACC. However, its low prevalence makes prospective treatment trials difficult to conduct. The ongoing RTOG 1008 randomized trial has been designed to investigate the efficacy of cisplatin-based POCRT in patients with salivary gland carcinomas and a pathologic stage of T3-4, N1-3, or surgical margins ≤ 1 mm. However, this trial aims to enroll patients with a wide range of histological subtypes (including adenocarcinoma, mucoepidermoid carcinoma, salivary duct carcinoma, acinic cell carcinoma, and SGACC) until a sample size of 120 cases is achieved. Although the results of this trial will be paramount for clarifying the feasibility of POCRT in patients with salivary gland carcinomas, it is unclear whether the findings will be generalizable to SGACC. We therefore believe that our multicenter study using propensity score matching may add valuable information on the clinical usefulness of adding concurrent chemotherapy to PORT for SGACC patients.

## Conclusions

The results from our study indicate that the addition of concurrent chemotherapy to PORT may increase LRC and ORPFS rates in SGACC patients, particularly in presence of stage III − IV disease, positive surgical margins, or PNI. Unfortunately, no statistically significant differences were observed in terms of DMFS, DFS, and OS. The occurrence of distant metastases remains a major clinical issue in patients bearing adverse prognostic factors. Consequently, further studies on the potential usefulness of concurrent chemotherapy and the clinical value of novel systemic agents for SGACC are urgently required.
